# Consumption of *Terminalia catappa* powder: modulation of lipid metabolism, reduction of cardiovascular risk, and hepatic protection in aged *Wistar* rats

**DOI:** 10.1007/s10522-026-10393-5

**Published:** 2026-03-02

**Authors:** Bruno Silva Dantas, Diego Elias Pereira, Natália Dantas Oliveira, Arielly Cristina Soares Oliveira, Larissa Maria Gomes Dutra, Josefa Dayse Lima Silva, Juliano Carlo Rufino Freitas, Raphaela Veloso Rodrigues Dantas, Carlos Eduardo Vasconcelos Oliveira, Ana Cristina Silveira Martins, Danilo Lima Dantas, Macilio Martins de Moraes, Vanessa Bordin Viera, Juliana Késsia Barbosa Soares

**Affiliations:** 1 Program in Natural Sciences and Biotechnology, Laboratory of Experimental Nutrition, Department of Nutrition, Federal University of Campina Grande, Cuité, PB Brasil; 2 Laboratory of Experimental Nutrition, Department of Nutrition, Federal University of Campina Grande, Cuité, CG Brazil; 3 Program of Food Science and Technology, Federal University of Paraíba, João Pessoa, PB Brazil; 4 Education and Health Center, Federal University of Campina Grande, Cuité, CG Brazil; 5 Laboratory of Organic Synthesis and Medicinal Chemistry, Department of Chemistry, Federal University of Campina Grande, Cuité, CG Brazil; 6 Department of Nutrition, Federal University of Campina Grande, Cuité, PB Brasil; 7 Department of Nutrition, Federal University of Paraíba, João Pessoa, JP Brazil; 8 Food Analysis Laboratory, Uniesp, Cabedelo, Brazil; 9 Department of Nutrition, Uni Nassau, Paulista, PE Brazil; 10 Graduate Program in Chemistry at the Federal Rural University of Pernambuco, Recife, PE Brazil; 11 Program in Natural Sciences and Biotechnology, Federal University of Campina Grande, Cuité, CG Brazil

**Keywords:** PUFAs, Antioxidant compounds, Lipid metabolism, Hepatic and cardiovascular health

## Abstract

**Abstract:**

The objective of this study was to evaluate the impact of *Terminalia catappa* powder consumption on biochemical, morphometric, cardiovascular risk, and hepatic markers in aged Wistar rats. Three groups were formed (n = 10): the control group (CG) was treated with distilled water, and the P500 and P1000 groups were treated with 500 and 1000 mg/kg of *Terminalia catappa* powder, respectively. Animal body weight and food intake were monitored weekly. At the end of the study, fecal samples were collected for cholesterol, triglycerides (TG), and fatty acid analysis. Additionally, murinometric and biochemical parameters were assessed. Hepatic tissue was harvested to evaluate cholesterol, TG, and malondialdehyde (MDA) levels. Food consumption and body weight showed no significant differences. In the P500 and P1000 groups, retroperitoneal fat weight was reduced, with P1000 also decreasing triglycerides (TG) and HDL levels. Both experimental groups registered lowered total cholesterol (TC), TG, and hepatic malondialdehyde (MDA) levels, with more pronounced effects in P1000, which also exhibited a higher proportion of unsaturated fatty acids. Fecal cholesterol increased in P1000, while fecal TG levels decreased in both treated groups. P1000 registered reduced cardiovascular and coronary risk indices and achieved the greatest reduction in MDA levels in coronary tissue. These results suggest that *Terminalia catappa* improves plasma and hepatic lipid metabolism, reduces body fat, and attenuates lipid peroxidation. Given its effects on cardiovascular risk factors, consumption of this fruit may contribute to reduced cardiovascular and coronary risks.

**Graphical Abstract:**

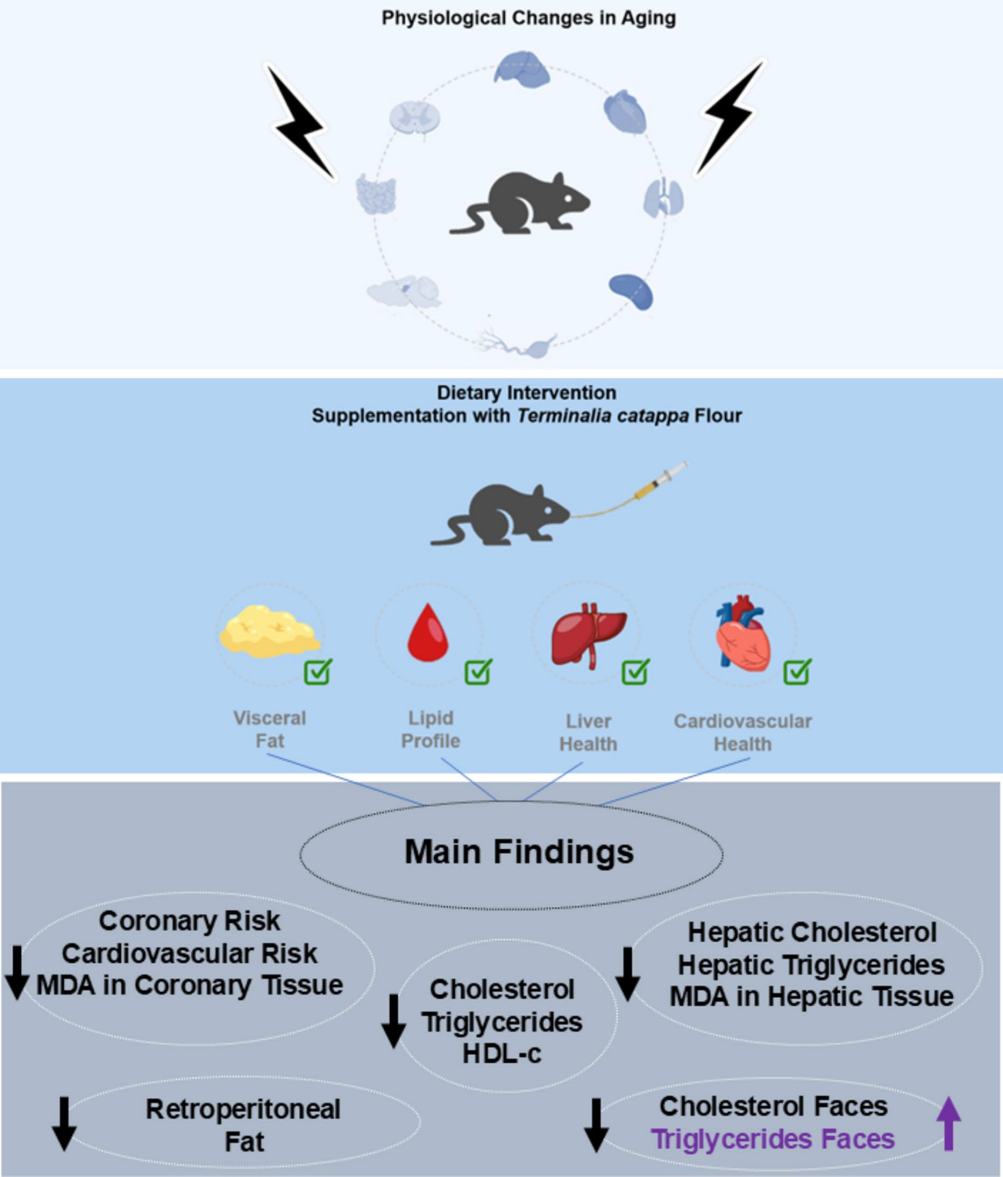

## Introduction

Life expectancy is projected to increase significantly over the coming decades, with estimates indicating that by 2050, approximately one-quarter of the global population will be 60 years or older(Dogra et al. 2022; Wise 2024). This demographic shift raises a critical question: Will the increase in life expectancy translate into healthier and more fulfilling lives, or will a rise in age-related diseases accompany it? (Menassa et al. 2023; Tenchov et al. 2024) Achieving healthier aging requires a proactive approach, emphasizing early interventions and promoting lifelong care through lifestyle modifications and dietary habits(Murphy et al. 2014).

A literature review indicated that the high consumption of Westernized diets, characterized by high amounts of sugars and saturated fats, is strongly associated with the development of non-communicable chronic diseases (NCDs) (Adolph and Tilg 2024). Research using experimental models has shown that high-fat diets promote increased oxidative stress in multiple organs,(Tursun et al. 2024; Yazıcı et al. 2023) which is one of the main underlying mechanisms in the pathogenesis of NCCDs. Oxidative stress has, in turn, been widely recognized as a crucial factor in modulating inflammatory and degenerative processes, directly implicating the deterioration of cellular and tissue function(Jomova et al. 2023; Hajam et al. 2022; Behl et al. 2021a).

On the other hand, the bioactive compounds present in plant matrices play a crucial role in mitigating oxidative stress, reducing inflammatory processes, and promoting cellular regeneration (Varesi et al. 2022; Mucha et al. 2021; Zhou et al. 2024). Some of these compounds exhibit antioxidant activity, directly neutralizing excess free radicals in the body and contributing to increased activity of the endogenous antioxidant system (Halliwell 2024).

Among the natural sources of antioxidants is the fruit of *Terminalia catappa,* which is native to tropical and subtropical regions and belongs to the Combretaceae family (Ramanan et al. 2025). It contains a range of antioxidant compounds, such as gallic acid, ellagic acid, quercetin, and their derivatives, as well as tannins and anthocyanins, which give the fruit a vibrant pigmentation and significant therapeutic potential (Oliveira et al. 2024).

In addition to antioxidants, *Terminalia catappa* also contains a lipid profile primarily composed of unsaturated fatty acids, known for their benefits related to fat metabolism, inflammation reduction, and improved cardiovascular health (Tabansi et al. 2023). Research involving rodent models has highlighted the bioactive potential of *Terminalia catappa* by correlating its bioactive compounds with the reduction of oxidative stress and inflammation (Iheagwam et al. 2021). Regulation of glucose metabolism (Iheagwam et al. 2022) and prevention of chronic diseases (Divya et al. 2019). However, none of this research has sought to evaluate the impact of *Terminalia catappa* consumption on the metabolism and health of aged rats.

Aging is inherently associated with a series of physiological changes that increase the risk of metabolic disturbances, even in the absence of dietary excesses such as high-fat intake. Age-related anomalies include impaired lipid metabolism, increased oxidative stress, chronic low-grade inflammation, and cardio-hepatic dysfunction, which collectively contribute to the development of metabolic syndromes and other non-communicable chronic diseases (Guo et al. 2022; He et al. 2024). Unlike studies that employ high-fat diets to induce metabolic damage, using aged animals allows for a more realistic and translational approach to evaluate interventions targeting naturally occurring senescence-related changes. In this context, *Terminalia catappa* powder represents a promising dietary strategy due to its antioxidant and anti-inflammatory properties, making it particularly relevant for the elderly population (Huang et al. 2018; Paik et al. 2024).

Given the urgent need for interventions that improve the quality of life for the elderly, we hypothesize that the incorporation of *Terminalia catappa* powder into the diet may reduce hepatic oxidative stress and improve important biochemical parameters during this stage of life. Therefore, the objective of this study was to evaluate the impact of *Terminalia catappa* powder consumption on biochemical parameters, morphometric measurements, cardiovascular risk, and hepatic markers in aged Wistar rats.

## Material and methods

### Terminalia catappa

The fruits of *Terminalia catappa* used in these experiments belong to the Combretaceae family. They were collected in the city of Cuité/PB, Brazil: (Latitude: -6.48173, Longitude: -36.1496; 6° 28′ 54″ South, 36° 8′ 59″ West. The species was deposited in the CES/UFCG Herbarium, with record 2893. To produce the *Terminalia catappa* powder, the fruits were manually depulped pulped before the drying process. The pulp was then spread onto stainless steel trays and dried in a forced-air circulation oven (Biopar, Model S480 AD, Porto Alegre, RS, Brazil) at 50 ± 1 °C for 48 h. After drying, the material was ground using a blender and subsequently sieved through a 0.5 mm mesh to ensure uniform particle size. The resulting powder was weighed, vacuum-sealed in sterile polypropylene bags, and stored at room temperature (23 ± 1 °C) until further analysis.

### Analysis physical–chemical

Physical–chemical analyses were performed on the *Terminalia catappa* powder. Water activity analysis was carried out using an AQUALAB device (DECAGON, Model AQUALAB 4TE, USA). The pH was determined using a digital pH meter (GEHAKA, model PG1800, São Paulo—SP, Brazil). Ash content was quantified by incineration in a muffle furnace (JUNG, Model 0612, Blumenau—SP, Brazil) stabilized at 550 °C. Humidity was determined by oven drying (Medclave, Model n° 4, Brazil), stabilized at 105 °C. Acidity was determined by titration according to the *Association of Official Analytical Chemists* – AOAC (Methods and of Analysis, 22nd Edition 2023). Lipids were determined by the Folch, Less and Stanley method, and the fatty acid profile was determined using the transesterification methodology of Hartman and Lago (1973) (Folch et al. 1957; Hartman and Lago 1973). Total insoluble and soluble fiber contents were ascertained using an enzymatic–gravimetric method.

### Analysis of antioxidant compounds

#### Extraction

*Terminalia catappa* powder constituents were extracted with an 80% methanol solution and evaluated for ABTS• removal capacity, ferric-reducing activity (FRAP), flavonoids, and total phenolics. The *Terminalia catappa* powder (1 g) was placed in a test tube and then 10 mL of solvent was added. The test tube was left at room temperature for 24 h, and after filtration, the volume was completed to 10 mL with extraction solvent and stored at 18 °C until analysis. All extractions were performed in triplicate.

#### Total polyphenol content

To measure the total phenolic compounds present in the sample, we used the methodology described by Liu et al. (2002) with minor adaptations (Liu et al. 2022). The absorbance of the extract was compared with a standard gallic acid curve to estimate the concentration of phenolic compounds in the sample. Results were expressed in milligrams equivalent to gallic acid/100 g of sample (mg EAG/100 g).

#### Total flavonoid content

A colorimetric assay developed by Zhishen et al. (1999) measured the total flavonoid content (Zhishen et al. 1999). To estimate the concentration of flavonoid contents in the sample, the extract’s absorbance was compared with a catechin standard curve. The total flavonoid content was expressed in mg equivalent to catechin/100 g of sample (mg EC/100 g).

#### FRAP assay

The FRAP method was performed according to Benzie and Strain, (1999), with modifications proposed by Rockenbach et al. (2011) (Benzie and Strain 1999; Rockenbach et al. 2011). The FRAP solution was used as a reference reagent and the absorbance was read in nm. Results were expressed as µmol trolox equivalents per gram sample (µmol TE/g^−1^).

#### ABTS assay

The ABTS method was performed by the methodology described by Surveswaran et al. (2007) (Surveswaran et al. 2007), with modifications. Results were expressed as µmol Trolox equivalents per gram of sample (µmol TE/g^−1^). Where A_0_ is the absorbance of the control. The effective concentration presented 50% radical inhibition activity (IC_50_), expressed in mg extract/mL, which was determined from the graph of the free radical scavenging activity (%) against the extract concentration (Table 1).

**Table 1:** Physicochemical characteristics and antioxidant potential of *Terminalia catappa* flour

Parameters	**CP**
Humitidy	14.12 ± 0.18
Lipids	1.38 ± 0.29
Ashes	5.92 ± 0.02
Acids	2.27 ± 0.02
aw	0.52 ± 0.00
pH	4.2 ± 0.08
Dietary fiber (g/100 g)	
Insoluble dietary fiber	31.45 ± 0.10
Soluble dietary fiber	2.32 ± 0.31
Total dietary fiber	33.77 ± 0.41
Antioxidant potencial	
Total phenolics (mg GAE)	106.6 ± 0.01
Total flavonoids (mg CE/100 g)	4.6 ± 0.03
FRAP (μmol TEAC/100 g)	2.07 ± 0.02
ABTS (μmol TEAC/100 g)	8.64 ± 0.00

Data are expressed as mean ± SD, n = 3; *GAE* Gallic acid equivalent, *CE* Catechin equivalent, *TE* Trolox equivalent

### Toxicological Study

The toxicological study was conducted using an adult Artemia salina Leach (A. salina). To induce hatching, 0.1 g of A. salina cysts were used, which were incubated in a rectangular glass container containing artificial saline water at a concentration of 38 g/L. The average temperature was maintained at approximately 28 °C, with constant illumination provided by a 40 W incandescent lamp for 24 h, as described by Meyer et al. (1982) (Meyer et al. 1982).

In the toxicological study of the extract, a stock solution was prepared and diluted to obtain minimum concentrations of 3250, 3000, 2000, 1750, 1500, 1250, 1000, 500, and 100 μg/mL. The different concentrations of the extract were distributed in test tubes containing exactly 10 A. salina per tube, and the study was carried out in triplicate for each concentration. Positive and negative tests were also carried out for comparison. After 24 h of exposure to the extract, a count was performed to determine the number of dead A. salina. The median lethal concentration (LC50), which is the concentration resulting in 50% lethality, was determined. Larvae that did not exhibit any normal movement within approximately 10 s of observation were considered dead.

### Animals, experimental design, and procedures

All of the experimental methods were previously approved by the Ethics Committee for Animal Use—CEUA of UFCG—Certification No. 53–2020, in compliance with the standards established by the National Council for the Control of Animal Experimentation (CONCEA, Brazil), under Law No. 11,794 /2008 (Arouca Law), and with the guidelines for in vivo experiments of the Animal Research: Reporting of In Vivo Experiments (ARRIVE) 2.0 (Sert et al. 2020). The *Terminalia catappa* used for the production diet administered to animals was registered in SisGen; protocol No. A92B6F0 (see attachment). Thirty male Wistar strains, aged 18 months and weighing 350 ± 20 g from the Federal University of Pernambuco were used. The animals were housed in individual polypropylene cages (60 cm long, 50 cm wide, and 22 cm high), and kept under standard laboratory conditions (temperature 22 ± 1 °C, humidity 55 ± 5%, light/dark cycle of 12/12 h—artificial light from 6:00 to 18:00). Four groups were formed: Adult Control Group (CGad): adults rats supplemented with distilled water, (n = 11); Aged Control Group (CGa): aged rats supplemented with distilled water (n = 11); P500: supplemented with 500 mg of Terminalia catappa powder /kg of body weight; and P1000: supplemented with 1000 of Terminalia catappa powder /kg of body weight. Gavage treatment was administered for 35 days, during which all animals were provided with standard feed Labina® (Presence Purina, São Paulo, Brazil) and water ad libitum. The composition of the commercial rodent chow included a minimum of 23% crude protein, 4% ether extract (fat), a maximum of 7% crude fiber, and a maximum of 10% ash content. The formulation also provided calcium ranging from 0.8 to 1.2%, a minimum of 0.4% phosphorus, and was supplemented with essential vitamins (A, D₃, E, K, and B-complex) and trace minerals such as iron, zinc, manganese, copper, and selenium.

Dosage of Terminalia *catappa* powder was based on previous studies. Behl, Valpadian, and Kotwani (2021) (Behl et al. 2021b) evaluated the effects of *Terminalia catappa* fruit extract in doses of 20, 30, and 40 mg/kg in a streptozotocin-induced diabetic retinopathy model in rats. In addition, Naitik, Prakash, Kotrsha, and Rao (2012) reported antitumor and lipid-lowering effects following the administration of *T. catappa* in doses of 250 and 500 mg/kg for 20 days in Wistar albino rats (Naitik et al. 2012).

Throughout the experimental period, during the light phase, body weight and feed intake were recorded weekly using a Balmak® digital scale (Model: ELP-10, Santa Bárbara do Oeste/SP, Brazil; range: 20–10,000 g). Prior to anesthesia, fecal samples were collected for the analysis of total cholesterol and triglycerides. Following anesthesia, the animals underwent murinometric evaluation, which included measurements of naso-anal length, abdominal and thoracic circumferences, and final body weight.

**Fig. 1:**
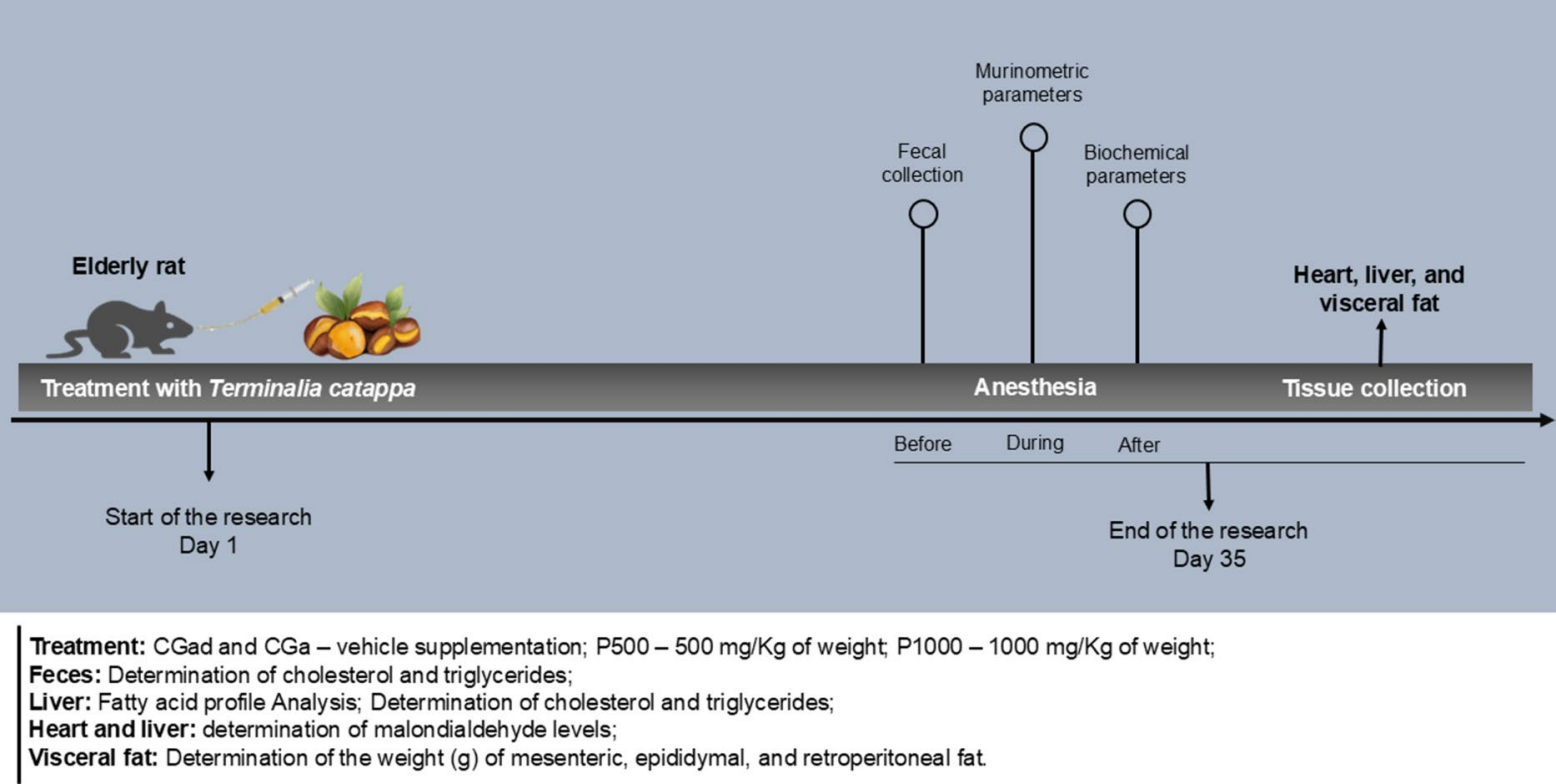
Experimental Protocol. Timeline of the experimental procedure (days) involving Wistar rats treated during adulthood (CGad) and the aging phase with distilled water (CGa), 500 mg/kg of *Terminalia catappa* powder (P500), and 1000 mg/kg of *Terminalia catappa* powder (P1000)

Blood was then collected via cardiac puncture for subsequent biochemical analyses. After euthanasia, organs of interest, including the liver, kidneys, heart, and carcass, were excised and weighed. Adipose tissues (mesenteric, epididymal, and retroperitoneal) were also dissected and weighed. The liver was sectioned: the right lobe was allocated for cholesterol and triglyceride quantification, whereas the left lobe was used for assessing lipid peroxidation (malondialdehyde, MDA) and fatty acid profile. The experimental protocol is detailed in Fig. 1.

### Blood collection and biochemical profile analyses

Blood was collected in tubes containing sodium fluoride used as an anticoagulant for plasma separation intended for glucose measurement. For the remaining biochemical analyses, the blood was collected in tubes without anticoagulant to allow serum separation. Subsequently, the samples were centrifuged (Bench Digital Centrifuge – NT 810, Novatecnica, Piracicaba – São Paulo, Brazil) at 1308 G for 15 min. Plasma was used for glucose analysis, while serum was used to measure total cholesterol, triglycerides, high-density lipoprotein (HDL), creatinine, urea, aspartate aminotransferase (AST), and alanine aminotransferase (ALT). The analyses were performed using enzymatic methods with a commercial kit from Labtest (Minas Gerais, Brazil), and readings were taken using a spectrophotometer (Kasuaki, model IL-226-NM-BI, Araucária, Brazil).

### Adiposity index (ADI), coronary risk index (CRI), and cardiovascular risk index (CVRI)

The coronary risk index (CRI) and cardiovascular risk index (CVRI) were determined using the equations: CRI = CTr/HDL; IRCV = TG/HDL, respectively (CTr = Cholesterol; TG = triglycerides) (Friedewald et al., 1972). The adiposity index (AI) was calculated using the formula: [body fat weight (epididymal + visceral + retroperitoneal)/body weight] × 100 (Nascimento et al. 2011).

### Hepatic and fecal cholesterol and triglycerides

The analysis of feces and hepatic lipids was performed following the method described by Folch, Less, and Stanley (1957) (Folch et al. 1957). After lipid extraction, cholesterol and triglyceride concentrations were determined. An enzymatic method, using the Labtest commercial kit (Minas Gerais, Brazil) and the reading were both carried out using a spectrophotometer (Kasuaki, model IL-226-NM-BI, Araucaria, Brazil).

### Determination of malondialdehyde in the liver and heart

At the end of the experiment, after 6 h of fasting, the animals were anesthetized with Ketamine Hydrochloride and Xilasin (1 ml/kg body weight) and were sacrificed. Then, the liver and heart tissues were removed to determine the content of MDA. To assess lipid peroxidation, MDA production was measured in an assay described by Esterbauer and Cheeseman, (1990) (Esterbauer and Cheeseman 1990). Tissue (5 samples per group) homogenates (T Tris–HCl 20 mm, 1:5 p/v) were centrifuged at 2500 g at 48 °C for 15 min, then were added to a 750 ml solution (1-Methyl-2-phenylindole 10.3 mm in acetonitrile + 225 ml HCl 37%) and the mixture was placed in a water bath and heated to 4 °C for 40 min. Next, it was centrifuged at 2500 g at 4 °C for 5 min. Absorbance was measured at 586 nm (Genesys 10 s UV–VIS, Thermo Fisher Scientific, Loughborough, UK). The concentration of MDA was expressed as nmol of MDA per gram of liver and heart tissues.

### Determination of the fatty acid profile in the liver

To determine the fatty acid profile of the liver, the lipid extract of the products was first obtained using the method of Folch et al. (1957) (Folch et al. 1957). From this extract, methyl esters were obtained by esterification following the methodology of Hartman and Lago, (1973) (Hartman and Lago 1973). The methyl esters were identified and quantified in a Ciola & Gregori Ltda gas chromatograph (model CG-Master), with a flame ionization detector. The chromatographic analysis used a polyethylene glycol column (Carbowax 20 M), with fused silica, 30 m long, 0.53 mm in diameter, and 0.25 μm thick stationary phase film. The vaporizer and detector temperatures were 150 °C and 200 °C, respectively. The oven program was 80 °C for 30 min, with an increase of 10 °C /min up to 180 °C. The mobile phase was hydrogen, with a flow rate of 5 mL/min. A volume of 1 μL was injected, with a split ratio of 1:25. The characterization of fatty acids was carried out by comparing the mass spectrum obtained with standards also injected into GC–MS.

## Statistical analysis

Results were expressed as mean ± standard error of the mean (SEM) and analyzed by analysis of variance (ANOVA), followed by Tukey’s post hoc test when significant differences between groups were detected (p < 0.05). Data normality was assessed using the Shapiro–Wilk test to ensure compliance with the assumptions of parametric analysis. All statistical analyses were performed using GraphPad Prism software, version 10.1.2.

## Results

### Toxicological study

The toxicity of the hydroethanolic extract of Terminalia catappa was analyzed at different concentrations, resulting in an LD50 of 2293 ppm, with a range of 2222 to 2366 ppm and a statistical confidence level of 95%.

### Food consumption and body weight

**Fig. 2:**
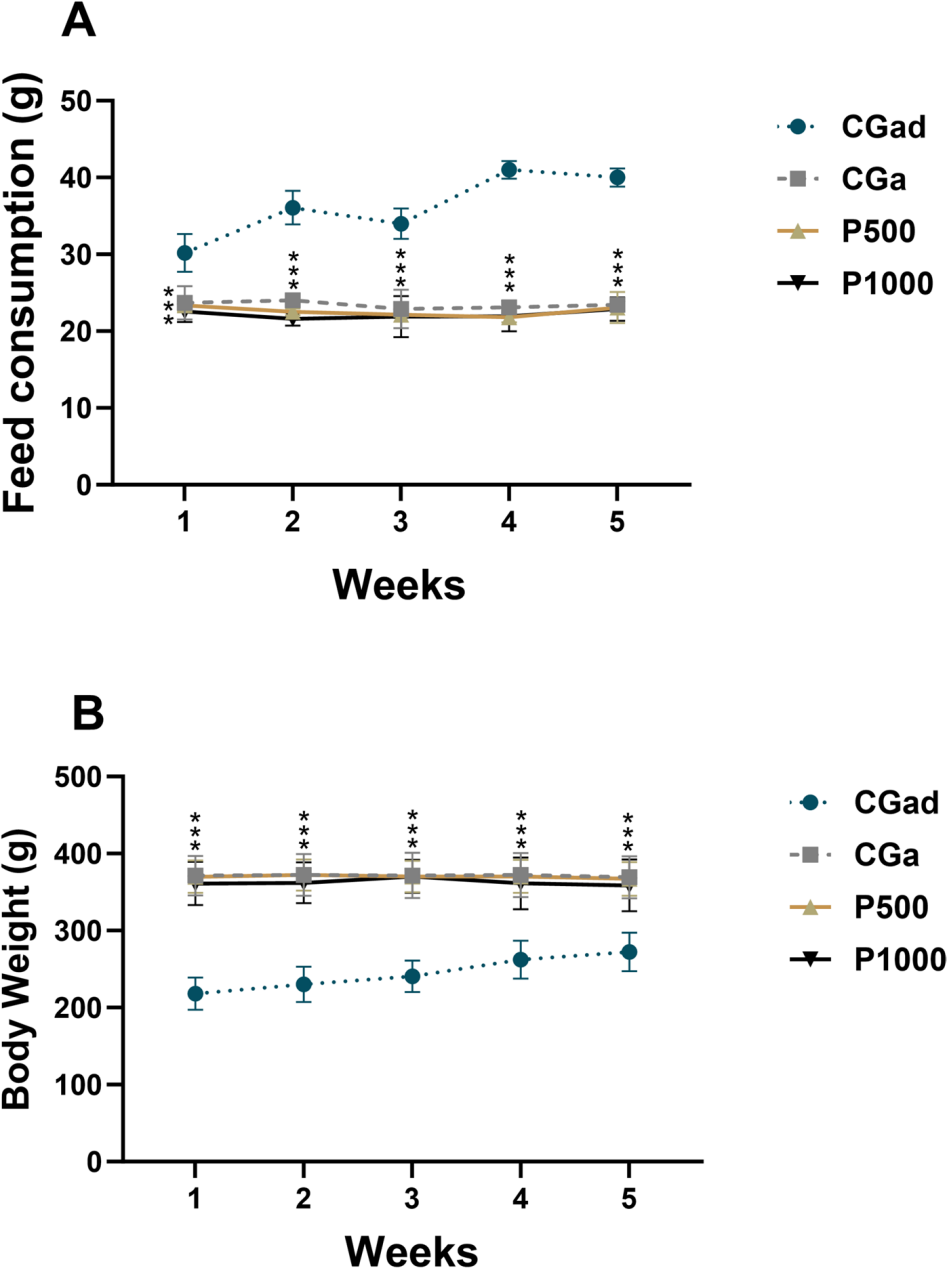
Food consumption and body weight of animals treated with 500 and 1000 mg/kg of *Terminalia catappa* powder during the aging phase compared to adult control group (CGad) and aged control group (CGa). Data are expressed as mean ± standard error. Adult Control Group (CGad) (n = 11); Aged Control Group (CGa); P500: supplemented with 500 mg of *Terminalia catappa* powder /kg of body weight (n = 11); P1000: supplemented with 1000 mg of *Terminalia catappa* powder /kg of body weight (n = 11). A—Food consumption (g); B—Body weight (g). Data were analyzed using One-Way ANOVA followed by Tukey’s post hoc test (p < 0.05). Different letters between bars signify differences between groups (p < 0.05)

The analysis of weekly feed intake and body weight over the 35-day treatment period revealed statistically significant differences between the adult control group and the other experimental groups (*p* < 0.05) (Fig. 2).

### Murinometric assessment

No statistically significant differences were observed between the experimental groups (P500 and P1000) and the aged control group with respect to naso-anal length, thoracic and abdominal circumference, organ weights, or carcass weight. Regarding adipose tissue, the treated groups exhibited reduced retroperitoneal fat mass compared to the aged control, yet higher values than those observed in the adult control group [F(3, 32) = 61.25, *p* < 0.0001]. For the remaining variables, significant differences were found only when comparing the adult control group with the aged and treated groups, which consistently showed higher values for naso-anal length, thoracic circumference, and abdominal circumference [F(3, 42) = 215.0, *p* < 0.0001; F(3, 42) = 91.43, *p* < 0.0001; F(3, 42) = 83.39, *p* < 0.0001, respectively]. Similar patterns were observed for liver, kidney, heart, and carcass weights [F(3, 42) = 30.20, *p* < 0.0001; F(3, 42) = 32.90, *p* < 0.0001; F(3, 42) = 63.45, *p* < 0.0001; F(3, 33) = 11.36, *p* < 0.0001]. Likewise, mesenteric and epididymal fat depots were significantly increased in the aged and treated groups compared to the adult control [F(3, 42) = 23.65, *p* < 0.0001; F(3, 39) = 27.43, *p* < 0.0001] (Table 2).

**Table 2:** Murinometric assessment of experimental groups

Parameters	CGad	CGa	P500	P1000
Naso-anal length (cm)	18.46 ± 0.37^a^	24.41 ± 0.83^b^	24.35 ± 0.57^b^	23.91 ± 0.92^b^
TC (cm)	11.25 ± 0.26^a^	14.86 ± 0.81^b^	14.86 ± 0.64^b^	14.23 ± 0.68^b^
AC (cm)	11.66 ± 0.35^a^	16.09 ± 1.02^b^	15.77 ± 0.82^b^	15.45 ± 0.99^b^
Organs and carcass weights (g)				
Liver weight	7.35 ± 0.59^a^	12.11 ± 1.60^b^	12.58 ± 1.42^b^	12.03 ± 2.33^b^
Kidney weight	1.53 ± 0.13^a^	2.39 ± 0.18^b^	2.38 ± 0.14^b^	2.38 ± 0.28^b^
Heart weight	0.78 ± 0.07^a^	1.23 ± 0.08^b^	1.24 ± 0.11^b^	1.24 ± 0.13^b^
Carcass weight	180 ± 26.47^a^	241.00 ± 23.65^b^	244.82 ± 18.93^b^	233.20 ± 30.84^b^
Visceral fats (g)				
Mesenteric fat	3.18 ± 0.47^a^	5.31 ± 1.52^b^	5.22 ± 1.52^b^	5.46 ± 1.15^b^
Epididymal fat	1.04 ± 0.14^a^	4.40 ± 1.53^b^	4.47 ± 1.17^b^	4.65 ± 1.31^b^
Retroperitoneal fat	1.19 ± 0.25^a^	6.35 ± 2.43^b^	5.74 ± 2.43^b^	6.33 ± 1.89^b^

Data are expressed as mean ± standard error. CGad: Adult Control Group (n = 11); GCa: Aged Control Group (n = 11); P500: supplemented with 500 mg of *Terminalia catappa* powder /kg of body weight(n = 11); P1000: supplemented with 1000 mg of *Terminalia catappa* powder /kg of body weight (n = 11). Data were analyzed using One-Way ANOVA followed by Tukey’s post hoc test. Different letters between bars signify differences between groups (p < 0.05)

### Blood collection and biochemical profile analyses

**Fig. 3:**
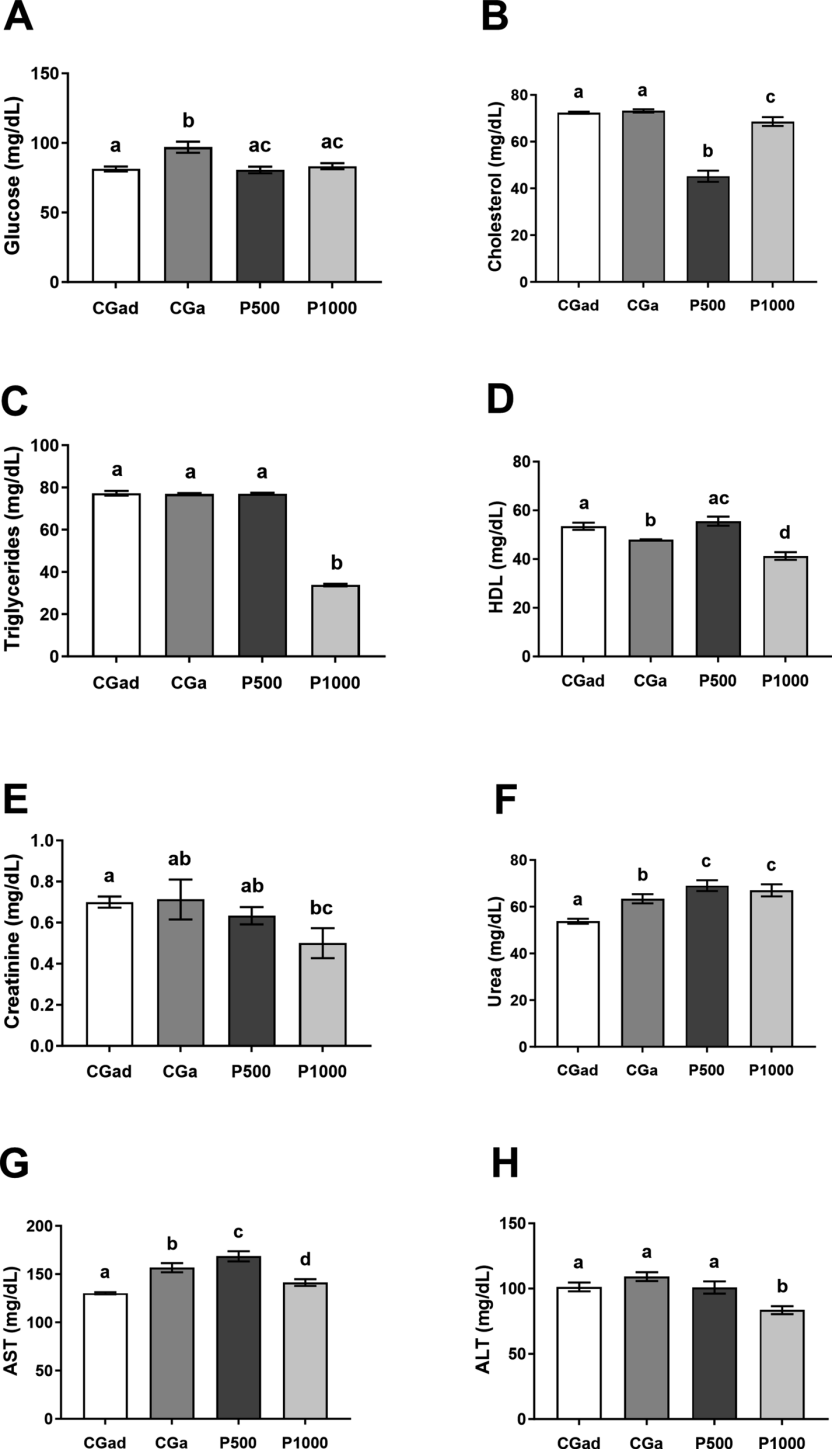
Biochemical parameters. Data are expressed as mean ± standard error. CGad: Adult Control Group (n = 11); GCa: Aged Control Group (n = 11); P500 (n = 11); P1000 (n = 11). Different letters between bars signify differences between groups (p < 0.05). A—Glucose (ml/dL); B—Cholesterol (ml/dL); C—Triglycerides (ml/dL); D—HDL Cholesterol (ml/dL); E—Creatinine (mg/dL); F—Urea (mg/dL); G—Aspartate aminotransferase (IU/L); H—Alanine aminotransferase (IU/L). Data were analyzed using One-Way ANOVA followed by Tukey’s post hoc test (p < 0.05). Different letters between bars signify differences between groups (p < 0.05)

Biochemical analyses revealed that fasting glucose levels were elevated in the aged control group (CGa) compared to the adult control group (CGad), while both P500 and P1000 groups exhibited reduced levels relative to CGa. However, no statistically significant differences were found between the experimental groups and CGad [F(3, 29) = 7.495; *p* = 0.0007] (Fig. 3A).

Total cholesterol levels were significantly reduced in the P500 and P1000 groups compared to both control groups (CGad and CGa), with the most pronounced decrease observed in P500 [F(3, 30) = 62.82; *p* < 0.0001] (Fig. 3B).

The P1000 group also showed a marked reduction in serum triglyceride levels when compared to all other groups [F(3, 26) = 1247; p < 0.0001] (Fig. 3C). Regarding HDL concentrations, the P500 group presented significantly higher levels than both CGa and P1000, reaching values comparable to those of the CGad group [F(3, 23) = 18.22; *p* < 0.0001] (Fig. 3D).

For serum creatinine, lower concentrations were observed in the P1000 group compared to CGad [F(3,24) = 1.919; *p* = 0.0108]. In contrast, urea levels were elevated in both P500 and P1000 groups relative to the control groups [F(3,32) = 11.03; *p* < 0.0001].

With respect to hepatic enzymes, AST levels were significantly higher in the P500 group compared to P1000 and both control groups [F(3,32) = 13.17; p < 0.0001]. Notably, AST concentrations were lower in P1000 than in CGa. ALT levels were significantly reduced in the P1000 group compared to all other groups, indicating a potential hepatoprotective effect [F(3,22) = 8.621; *p* = 0.0006].

### Adiposity index (ADI), coronary risk index (CRI), cardiovascular risk index (CVRI), and malondialdehyde levels in coronary tissue

**Fig. 4:**
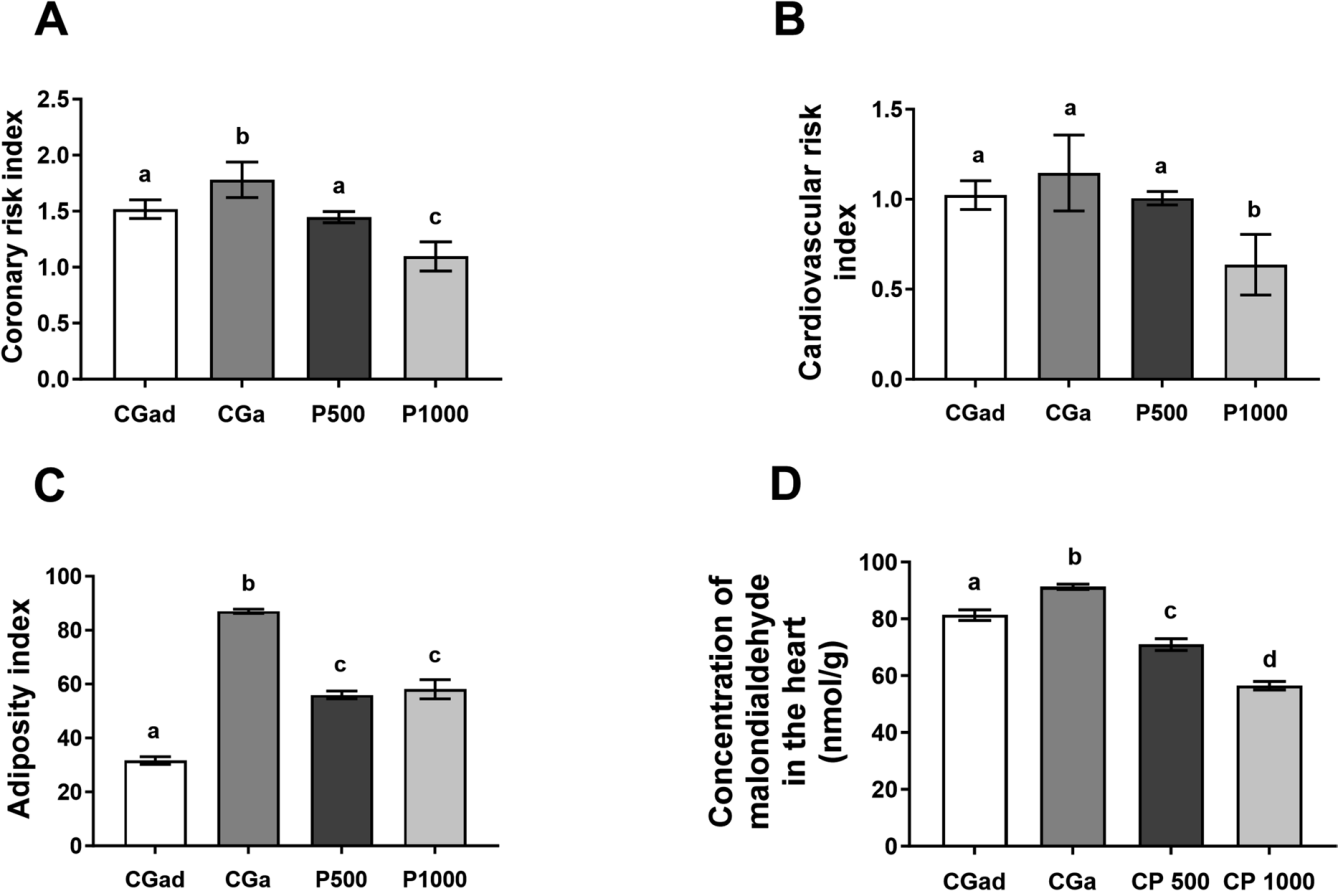
Adiposity index (ADI), Coronary risk index (CRI), Cardiovascular risk index (CVRI), and Malondialdehyde (MDA) levels in coronary tissue. A—Data are expressed as mean ± standard error. CGad: Adult Control Group (n = 11); GCa: Aged Control Group (n = 11); P500 (n = 11); P1000 (n = 11). Different letters between bars signify differences between groups (p < 0.05). A—Coronary risk index; B—Cardiovascular risk index; C—Adiposity index; D—Malondialdehyde (nmol/g). Data were analyzed using One-Way ANOVA followed by Tukey’s post hoc test (p < 0.05). Different letters between bars signify differences between groups (p < 0.05)

The coronary risk index was significantly reduced in the experimental groups compared to the aged control group (CGa). The group supplemented with 500 mg/kg of *Terminalia catappa* powder (P500) showed values comparable to those observed in the adult control group (CGad), whereas the group treated with 1000 mg/kg (P1000) exhibited a more pronounced reduction, with values lower than those of the CGad group. The cardiovascular risk index was significantly lower in the P1000 group compared to all other groups [F(3, 40) = 8.993, p < 0.0001; F(3, 33) = 4.488, p = 0.0095] (Fig. 4A, B).

When assessing the adiposity index, the experimental groups showed significant reductions compared to CGa; however, these values remained higher than those observed in the CGad group [F(3, 24) = 351.00, p < 0.0001] (Fig. 4C).

Additionally, cardiac tissue malondialdehyde (MDA) levels were significantly lower in the experimental groups compared to both CGa and CGad, with the P1000 group exhibiting the lowest levels among all groups [F(3, 20) = 97.81, p < 0.0001] (Fig. 4D).

### Hepatic cholesterol, triglycerides and malondialdehyde

**Fig. 5:**
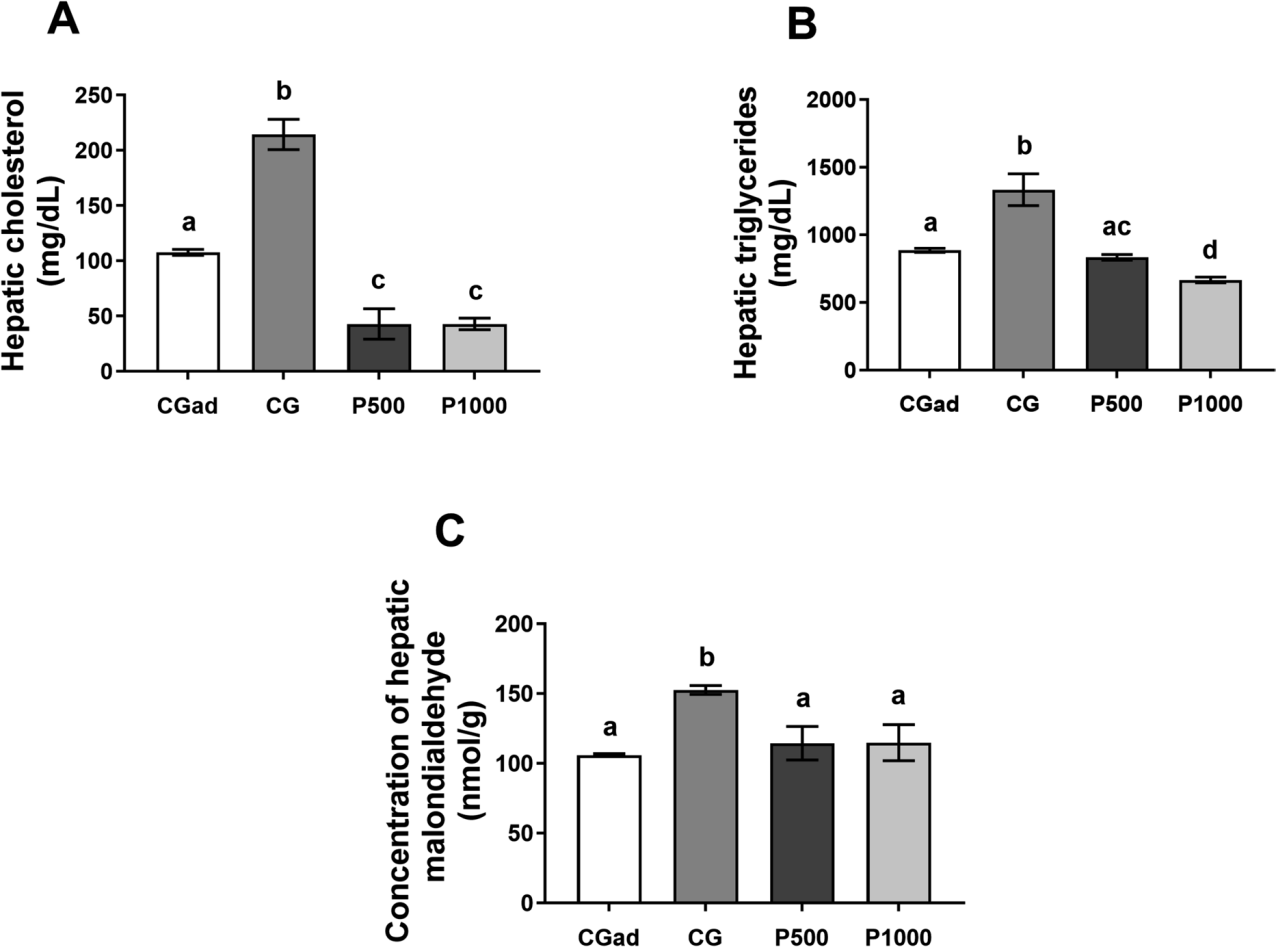
Hepatic cholesterol, triglycerides, and malondialdehyde (MDA). Data are expressed as mean ± standard error. CGad: Adult Control Group (n = 11); GCa: Aged Control Group (n = 11); P500 (n = 11); P1000 (n = 11). Different letters between bars signify differences between groups (p < 0.05). A—Hepatic cholesterol (mg/dL); B—Hepatic triglycerides (mg/dL); C—Malondialdehyde (nmol/g). Data were analyzed using One-Way ANOVA followed by Tukey’s post hoc test (p < 0.05). Different letters between bars signify differences between groups (p < 0.05)

Hepatic levels of cholesterol and triglycerides were elevated in aged control (CGa) animals compared to the adult control group (CGad). Treatment with 500 or 1000 mg of Terminalia catappa powder significantly reduced hepatic cholesterol concentrations in both control groups, although no significant difference was observed between the P500 and P1000 treatment groups [F (3. 20) = 62.99, p < 0.0001; F (3. 22) = 23.72, p < 0.0001) (Fig. 5A, B). In addition, CGa animals exhibited higher hepatic malondialdehyde (MDA) levels compared to CGad, while administration of 1000 mg/kg of *T. catappa* powder led to a significant reduction in MDA concentrations relative to the aged control group [F (3. 20) = 5.421, p = 0.0068] (Fig. 5C).

### Fecal cholesterol and triglycerides

**Fig. 6:**
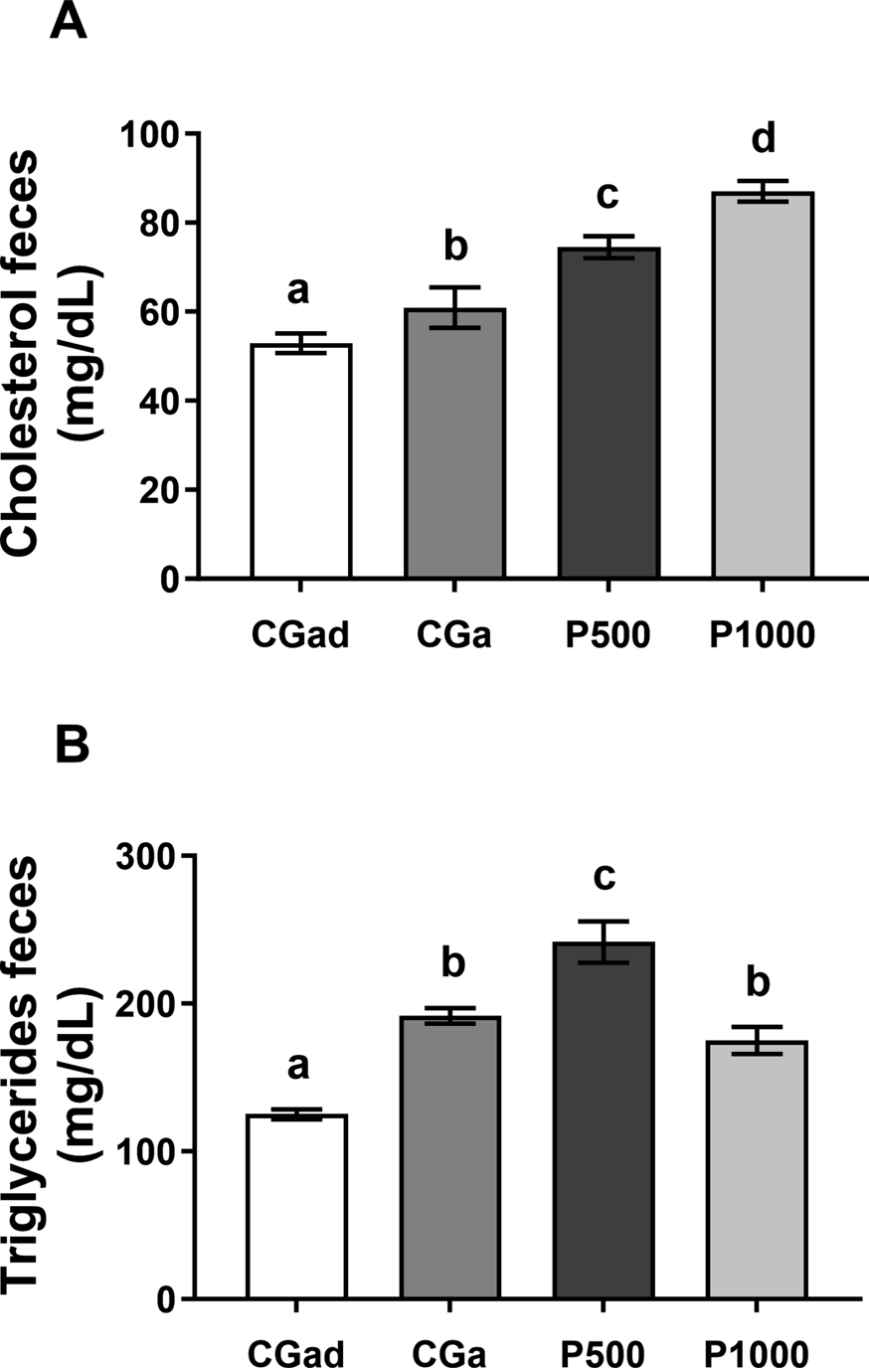
Fecal cholesterol and triglycerides. Data are expressed as mean ± standard error. CGad: Adult Control Group (n = 11); GCa: Aged Control Group (n = 11); P500 (n = 11); P1000 (n = 11). Different letters between bars signify differences between groups (p < 0.05). A—Fecal cholesterol (mg/dL); B—Fecal triglycerides (mg/dL). Data were analyzed using One-Way ANOVA followed by Tukey’s post hoc test (p < 0.05). Different letters between bars signify differences between groups (p < 0.05)

Fecal cholesterol analysis revealed no statistically significant difference between the adult (CGad) and aged (CGa) control groups. In contrast, the experimental groups (P500 and P1000) showed a significant increase in cholesterol excretion compared to the controls. Post hoc analysis further indicated a difference between the treated groups, with the highest elimination levels observed in the P1000 group [F (3. 22) = 26.84, p < 0.0001] (Fig. 6A). Regarding triglycerides, fecal excretion was elevated in CGa compared to CGad. The P500 group exhibited higher values than the aged control, while P1000 showed greater elimination relative to the adult control. Post hoc analysis revealed significantly lower triglyceride levels in the P1000 group compared to P500 [F (3. 22) = 36.00, *p* < 0.0001] (Fig. 6A).

### Liver fatty acid composition

The analysis of hepatic saturated fatty acids revealed an increase of 2.77% in the P500 group and 2.51% in the P1000 group compared to the aged control, whereas both groups showed reductions of 5.94% and 6.18%, respectively, relative to the adult control. Notably, the aged group exhibited higher levels of palmitic acid, which were progressively reduced following treatment. In contrast, stearic acid was more abundant in the adult control group compared to the aged and treated groups (*p* < 0.05).

Regarding monounsaturated fatty acids, there was a reduction of 24.38% in the P500 group and 25.53% in the P1000 group relative to the aged control, while both groups showed increases of 13.37% and 11.65%, respectively, when compared to the adult control. Oleic acid levels were significantly elevated in the aged control group and reduced in the treated groups, reaching values comparable to those observed in the adult control (*p* < 0.05).

For polyunsaturated fatty acids, increases of 4.52% and 10.89% were observed in the P500 and P1000 groups, respectively, compared to the aged control, alongside reductions of 15.32% and 10.16% relative to the adult control. Our data further demonstrated that aging markedly decreased the levels of arachidonic acid, DHA, and docosatetraenoic acid, while treatment led to partial or complete recovery of these fatty acids, particularly at the higher dose (*p* < 0.05) (Table 3).

**Table 3:** Fatty acid composition in the liver

Liver fatty acids		CGad	CGa	P500	P1000
SATURATED					
Lauric acid	C 12:0	0.06 ± 0.01	**-**	**-**	**-**
Palmitic acid	C16:0	17.5 ± 0.00^a^	21.11 ± 0.2^b^	20.04 ± 0.1^c^	18.33 ± 0.3^d^
Stearic acid	C18:0	23.97 ± 0.02^a^	16.72 ± 0.1^b^	18.90 ± 0.1^c^	20.59 ± 0.2^d^
Myristic acid	C14:0	0.22 ± 0.01^a^	0.32 ± 0.2^b^	0.27 ± 0.1^c^	0.19 ± 0.1^d^
⅀SFA		**41.69**	**38.15**	**39.21**	**39.11**
MONOUNSATURED					
Palmitoleic acid	C16:1w7	**0.02 ± 0.01**	**-**	**-**	**-**
Oleic acid	C18:1ω-9	8.13 ± 0.01^a^	12.22 ± 0.2^b^	9.24 ± 0.3^c^	9.10 ± 0.2^c^
⅀MUFA		**8.15**	**12.22**	**9.24**	**9.10**
POLYUNSATURED					
Linoleic acid	C18:2ω-6	21.15 ± 0.02^a^	23.42 ± 0.1^b^	21.04 ± 0.3^a^	19.10 ± 0.1^c^
Arachidonic acid	C20:4ω-6	26.09 ± 0.02^a^	18.28 ± 0.1^b^	21.98 ± 0.1^c^	25.62 ± 0.1^a^
Docosahexaenoic acid	C22:6 ω3	5.00 ± 0.01^a^	1.16 ± 0.1^b^	1.78 ± 0.1^b^	2.40 ± 0.1^c^
Docosatetraenoic acid	C22: 4ω-6	0.67 ± 0.01^a^	-	0.30 ± 0.1^b^	0.41 ± 0.1^c^
⅀PUFA		**52.91**	**42.86**	**44.8**	**47.53**

Data are expressed as mean ± standard error. CGad: Adult Control Group (n = 11); GCa: Aged Control Group (n = 11); P500: supplemented with 500 mg of *Terminalia catappa* powder /kg of body weight (n = 11); P1000: supplemented with 500 mg of *Terminalia catappa* powder /kg of body weight (n = 11). Data were analyzed using One-Way ANOVA followed by Tukey’s post hoc test. Different letters between bars means differences between groups (p < 0.05)

## Discussion

This study investigated, for the first time, the effects of consuming different concentrations of *Terminalia catappa* powder on the physical and biochemical parameters of aged Wistar rats. The results demonstrated that supplementation improved liver function by reducing oxidative stress, levels of transaminases and the deposition of triglycerides and cholesterol in the liver, thus reversing aging-induced damage. Additionally, a hypolipidemic effect was observed, particularly when the highest dose of *Terminalia catappa* powder was administered (P1000).

According to the results of our study on the physicochemical analysis of Terminalia catappa powder, along with findings from another study conducted by our laboratory, the nutritional and bioactive potential of the powder was confirmed. The analysis highlighted significant levels of proteins, carbohydrates, and dietary fibers, particularly cellulose and lignin, which contribute to digestive health, increased satiety, and reduced fat absorption. Furthermore, the lipid profile analysis revealed a predominance of unsaturated fatty acids over saturated ones, with emphasis on oleic acid, which is associated with cardiovascular benefits, and linoleic acid, an essential fatty acid for the human diet due to its role in regulating lipid metabolism, inflammation, and LDL levels. Additionally, the powder also contained significant levels of total flavonoids and phenolic compounds as gallic acid, ellagic acid, quercetin and its derivatives, which are widely recognized for their antioxidant and anti-inflammatory properties (Oliveira et al. 2024).

In light of this, we decided to evaluate the effects of consuming *Terminalia catappa* powder in vivo, considering doses of 500 and 1000 mg/kg of body weight. Regarding the toxicity of the doses used in the present study, our evaluation using Artemia salina larvae indicated an LD50 of 2293 ppm, well above what was tested on animals in the present research. According to Meyer et al. (1982), (Meyer et al. 1982) plant extracts are classified as non-toxic when their LC50 (lethal dose for 50% of organisms) is greater than 1000 ppm, while lower values are considered toxic. Sereno et al. (2024) (Sereno et al. 2023) investigated the toxicity of fresh and dehydrated leaf extract of Campomanesia xanthocarpa Berg and observed no toxicity, especially in the dehydrated material. Therefore, we can consider the dose used in the present research to be non-toxic and no clinical signs of toxicity were observed in the rats studied.

Despite the functional composition of the powder, our data did not indicate significant changes in food intake or body weight between the aged groups. Although we did not observe a significant difference in the body weight of the animals, our results demonstrated that supplementation had a selective impact on visceral fat deposits, with a significant reduction in retroperitoneal fat in both experimental groups (P500 and P1000) compared to the aged control group, while mesenteric and epididymal fat weights remained unchanged. This specific effect may be attributed to the presence of the unsaturated fatty acids oleic and linoleic acids found in the powder, which are known to regulate lipid metabolism by stimulating the mobilization of fats from adipose tissue and their mitochondrial oxidation through the modulation of Peroxisome Proliferator-Activated Receptors (PPARs) (Flachs et al. 2009; Shin 2022; Varga et al. 2011). However, body weight and visceral fat accumulation was greater when compared to CGad, which is common with increasing aging.

These results contrast with previous studies that link the consumption of dietary fiber sources to reduced body weight gain in rodents (Ji et al. 2021; Wang et al. 2021). Thus, for the older rats, the polyunsaturated fatty acids and phenolic compounds present in *Terminalia catappa* powder were not able to regulate lipid metabolism by stimulating the mobilization of fats from adipose tissue and their mitochondrial oxidation through the modulation of Peroxisome Proliferator-Activated Receptors (PPARs) (Flachs et al. 2009; Shin 2022; Varga et al. 2011).

Upon analyzing the concentrations of cholesterol and triglycerides in the feces of the animals, we indeed observed a higher fecal excretion of cholesterol in both the P500 and P1000 groups. This effect can be explained by the ability of fibers and phenolic compounds to bind to bile acids in the intestinal lumen, thereby reducing their reabsorption and consequently increasing the fecal elimination of cholesterol (Liu et al. 2023; Joyce et al. 2019). We observed similar values in the fecal elimination of triglycerides of the P1000 rats compared to aged control group and increased excretion compared to the CGad. This increase may indicate lower efficiency in the absorption and/or utilization of triglycerides in the intestinal tract or an influence of the bioactive compounds on the digestion and absorption of fats. The opposite to our results was found by Sugiyama et al. (2007), which suggests that phenolic compounds can interact with digestive enzymes, such as lipases, thereby reducing the release of triglycerides for fecal excretion (Sugiyama et al. 2007).

Our results also revealed significant data regarding cardiovascular health markers, particularly in the P1000 group, which demonstrated a considerably improved lipid profile. Total cholesterol (P500 and P1000) and triglyceride levels (P1000) were significantly reduced compared to the CGcad and CGa, supporting recent evidence which indicates that a decrease in these lipids is associated with a reduced atherosclerotic risk (Mach et al. 2020). Although high-density lipoprotein levels showed a decline in the P1000 group, the reduction in coronary and cardiovascular risk indices underscores the importance of a multifactorial approach in assessing cardiovascular risk. This assessment should not only include lipid parameters but also inflammatory and metabolic factors (Tian et al. 2022).

Furthermore, it is important to note that in the evaluation of malondialdehyde levels in coronary tissue, there was a significant reduction of this compound in the P500 and P1000 groups compared to both control groups. This finding indicates that the potential role of antioxidant mechanisms related to oxidative stress is associated with atherosclerotic processes (Młynarska et al. 2024). Malondialdehyde, a marker of lipid peroxidation, is closely linked to oxidative damage in cell membranes, and its reduction suggests a relevant protective effect that complements the improvements observed in lipid parameters (Mas-Bargues et al. 2021; Lankin et al. 2022; Seenak et al. 2021). These findings reinforce the hypothesis that cardiac oxidative damage caused by aging can be reduced by consuming *Terminalia catappa* powder.

The analysis of hepatic fatty acid profiles revealed that aging markedly impairs lipid metabolism, characterized by a substantial reduction in polyunsaturated fatty acids (PUFAs) and the accumulation of saturated and monounsaturated fatty acids. In the present study, the aged control group exhibited elevated hepatic levels of palmitic and oleic acids, lipid species strongly associated with the activation of pro-inflammatory pathways, oxidative stress, and hepatic steatosis (Ceja-Galicia et al. 2025; Park et al. 2020; Patel et al. 2016). These age-related alterations were partially or fully attenuated in animals supplemented with *Terminalia catappa*, particularly at the highest dose (P1000), which exerted the most pronounced modulatory effect on the hepatic lipid profile. Although a slight increase in total saturated fatty acids was observed in the treated groups, palmitic acid levels were significantly reduced, suggesting a beneficial adaptive response to the metabolic stress associated with senescence.

The reduction and normalization of oleic acid levels following treatment also indicates a possible suppression of lipogenic pathways typically upregulated during aging (Castillo et al. 2024; Mutlu et al. 2021). Concurrently, the significant increase in PUFAs, particularly arachidonic acid and docosahexaenoic acid (DHA), in the P1000 group suggests a functional remodeling of hepatic lipid composition. These long-chain fatty acids, especially those from the omega-3 and omega-6 series, are well known for their anti-inflammatory properties, ability to restore membrane fluidity, and support of mitochondrial function, thereby contributing to metabolic integrity and the prevention of hepatic inflammatory damage (El-Mowafy et al. 2022; Saleh et al. 2022; Zhang et al. 2024; Šmíd et al. 2023).

These results demonstrate that *Terminalia catappa* powder not only improves liver health by reducing lipid accumulation but also promotes a less oxidative cellular environment, with potential implications for the prevention of diet-related liver conditions, such as non-alcoholic fatty liver disease (NAFLD). Despite these promising results, future translational research involving elderly individuals is recommended. It is important to note that, according to Nair and Jacob (2016), the doses of powder used in our study with rodents (500 and 1000 mg/kg) are equivalent to approximately 7.14 and 14.29 mg/kg in elderly humans.

Despite the relevance of the results, some limitations of this study should be acknowledged. Key inflammatory markers, including TNF-α, IL-6, C-reactive protein (CRP), and the NF-κB signaling pathway, were not assessed. These parameters could have provided additional evidence regarding the anti-inflammatory effects of *Terminalia catappa*. Furthermore, measurements of endogenous antioxidant defenses, such as catalase, superoxide dismutase (SOD), and reduced glutathione (GSH), were not conducted. Inclusion of these markers in future studies would allow for a more comprehensive understanding of the antioxidant mechanisms involved and further clarify the protective role of *Terminalia catappa* against hepatic oxidative stress and inflammation, helping to understand pharmacokinetics in aged models.

Moreover, no mechanistic analyses were performed to investigate intracellular pathways potentially modulated by *Terminalia catappa*. Future research should consider evaluating the activation of AMP-activated protein kinase (AMPK) and the modulation of peroxisome proliferator-activated receptors (PPARs), which are critical regulators of energy metabolism, lipid homeostasis, and inflammation. Understanding whether *Terminalia catappa* can influence these molecular targets would provide valuable insights into its nutraceutical potential, particularly in the context of aging.

## Conclusions

This study highlights the potential benefits of *Terminalia catappa* powder on liver function and lipid metabolism modulation in aged Wistar rats and reversal of damage caused by aging, a fact evidenced when we compare the data from adult rats. Although there were no significant changes in body weight or food intake, supplementation reduced retroperitoneal fat deposits and improved lipid profiles, particularly at the higher dose (P1000). The reductions in total cholesterol, triglycerides, and hepatic enzyme activities, along with the increased fecal excretion of cholesterol and triglycerides, suggest a beneficial effect on metabolic health and protection against fat accumulation in the liver.

The fatty acid analysis showed an increase in polyunsaturated fatty acids and a decrease in saturated and monounsaturated fatty acids in the livers of the treated groups, corroborating the importance of bioactive compounds in modulating lipid homeostasis. Thus, *Terminalia catappa* powder not only reduces lipid accumulation but also promotes a less oxidative cellular environment, which may help prevent diet-related liver diseases, such as non-alcoholic fatty liver disease.

## Data Availability

No datasets were generated or analysed during the current study.
